# Delayed Recognition, Surgical Dilemmas, and Complications: A Case Report of Acute-on-Chronic Subdural Hematoma With Ischemic Stroke

**DOI:** 10.7759/cureus.50610

**Published:** 2023-12-15

**Authors:** Noor Al-Thawwab, Dunya Alfaraj, Ashwaq Alenezi

**Affiliations:** 1 General Practice, Imam Abdulrahman Bin Faisal University, Dammam, SAU; 2 Emergency Department, Imam Abdulrahman Bin Faisal University, King Fahad University Hospital, Dammam, SAU; 3 Department of Pediatrics, Imam Abdulrahman Bin Faisal University, King Fahad University Hospital, Dammam, SAU

**Keywords:** embolization of middle meningeal artery, hypertension, head trauma, chronic subdural hematoma (csdh), acute on chronic subdural hematoma

## Abstract

Acute-on-chronic subdural hematoma (SDH) is a new hemorrhage on a preexisting hematoma in the space between the dura mater and the arachnoid layer. Although chronic SDH is common, acute-on-chronic SDH is not. Herein, we present a case of a 70-year-old male with ischemic heart disease, diabetes mellitus, and hypertension who presented with worsening headaches for the past three days, associated with gait imbalance and dizziness. The patient was vitally stable on examination with a Glasgow Coma Scale/Score (GCS) of 15/15; his pupils were reactive bilaterally; and his neurological examination was unremarkable. Non-contrast computed tomography (CT) of the head yielded acute and chronic SDH. The patient was initially managed by embolization of the middle meningeal artery (EMMA), but one day later he developed a stroke. Hence, thrombectomy and burr hole craniotomy were performed to manage the stroke and evacuate the chronic subdural hematoma (CSDH). This paper presents this case as uncommon acute on chronic SDH presented with headache and vertigo, treated with embolization and with complications of stroke and pulmonary embolism.

## Introduction

Subdural hematoma (SDH) is defined as the accumulation of blood between the dura mater and the arachnoid layer [1]. SDH is categorized into acute, subacute, and chronic based on the time of presentation, with acute presentation occurring within three days and chronic presentation occurring after 20 days [1]. CSDH is common, especially in the elderly population, with trauma being the most frequent risk factor [2]. Hypertension, bleeding diathesis, and the use of anticoagulants and antiplatelets are considered important risk factors as well [3,4]. The presentation of CSDH varies; patients may be asymptomatic or may experience a variety of symptoms such as headache, disorientation, vertigo, or seizures [2]. The definitive management technique is surgical management; however, some patients may only be observed closely or receive medical management [1,2]. Surgical management includes a twist-drill craniotomy and preferable burr hole evacuation due to fewer complications and a low chance of recurrence [2]. Other less invasive options include embolization of the middle meningeal artery (EMMA) [2]. Although EMMA is a safe procedure, it has some complications. Treatment failure is the most common difficulty, which might worsen the hematoma, and other complications may include neurological complications such as stroke, blindness, facial nerve palsy, aphasia, and/or pulmonary embolism (PE) [5,6]. Although acute-on-chronic SDH is uncommon, it has been reported in the literature [4,7,8]. Here, we describe a case of acute-on-chronic SDH presented with headache and vertigo, treated with embolization and complicated by stroke and PE.

## Case presentation

A 70-year-old male with type 2 diabetes mellitus, hypertension, and ischemic heart disease underwent percutaneous coronary intervention (PCI) three times. He presented to the emergency room (ER) complaining of a worsening headache for the last three days. The patient reported that he had been suffering from a headache for one month, which had become more severe over the last three days. The pain was associated with dizziness and an unsteady gait. The patient denied a history of abnormal movement, a change in the level of consciousness, weakness or numbness, nausea, or vomiting. There was no history of head trauma.

The patient was on metformin for diabetes and lisinopril for hypertension and stopped taking aspirin three months ago. The patient described a history of vertigo one month ago when moving his head, for which he visited the ER. The physical examination was remarkable for high blood pressure only, and investigations were unremarkable. After stabilization, the patient was discharged from the ER.

On examination, the patient looked well. He was conscious, alert, and oriented. His vitals were as follows: pulse rate, 138; blood pressure, 112/89 mmHg; oxygen saturation on room air, 99%. Upon neurological examination, the patient scored 15 out of 15 on the Glasgow Coma Scale/Score (GCS), with pupils three millimeters reactive bilaterally; cranial nerve examination was unremarkable; power was 5/5; and his coordination was intact.

**Figure 1:**
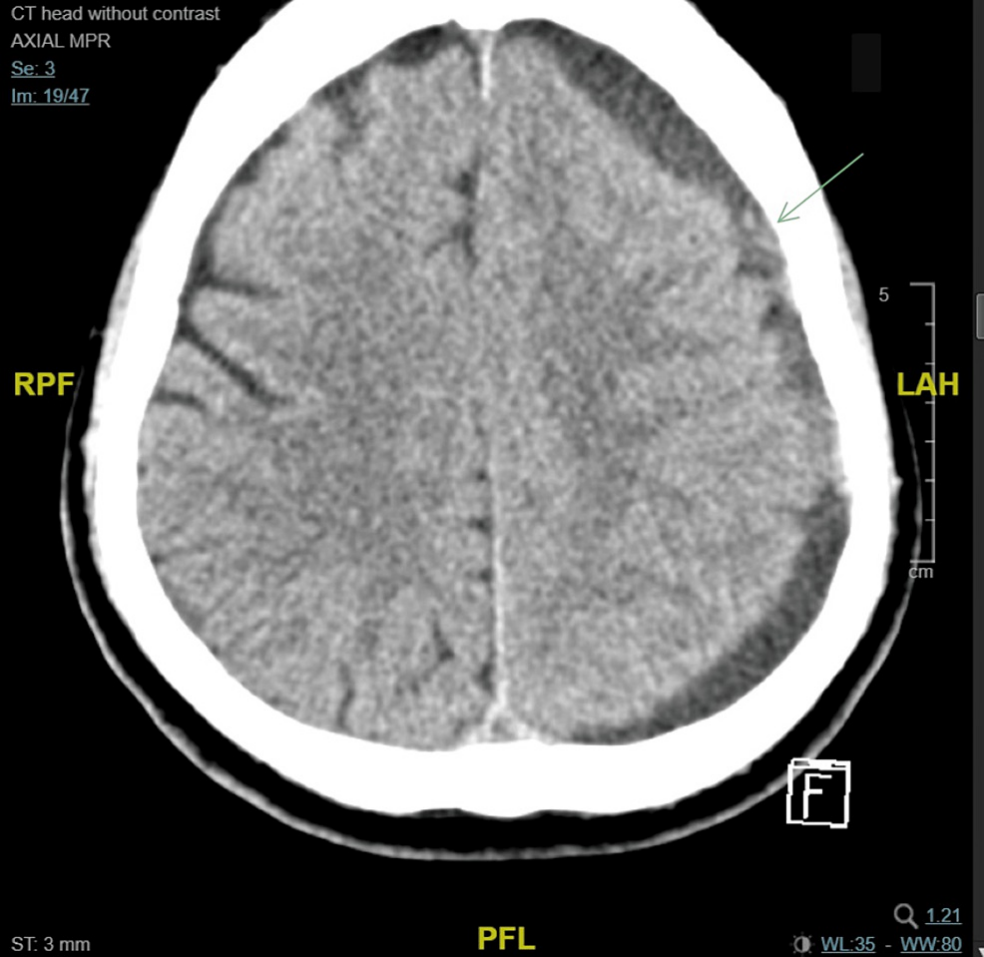
Non-contrast CT of the patient’s head indicates left-side subdural hygroma with internal different ages of extra-axial hemorrhage associated with midline shift as well as mass effect on the lateral ventricles.

Blood samples were obtained for laboratory investigations, including random blood glucose, complete blood count (CBC), liver and renal function tests, and coagulation profiles. The results were significant for decreased hemoglobin at about three months, which was 13.1 g/dL, and was 8.1 g/dL at presentation; otherwise, tests were within normal limits. Other investigations included an ECG and chest x-ray, which were normal. A non-contrast head CT was performed, which indicated left-side acute on chronic subdural hematoma with mass effect (Figure 1).

**Figure 2:**
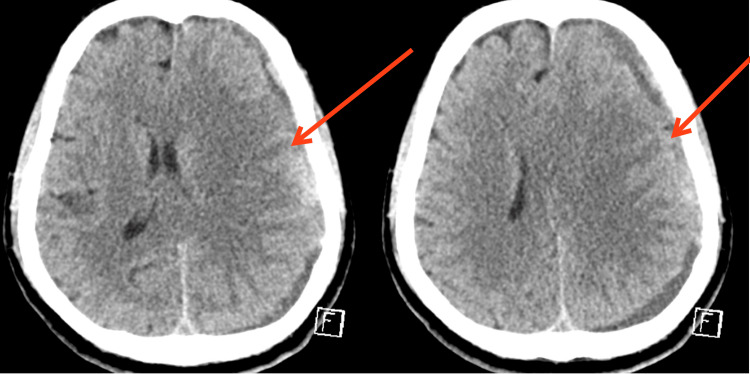
A non-contrast CT shows ischemic changes involving left MCA territory as well as progression of SDH, post EMMA. CT: computed tomography, MCA: middle cerebral artery, SDH: subdural hematoma, EMMA: embolization of middle meningeal artery.

The patient was admitted to the ward with close monitoring of his vital signs. Moreover, he received two units of packed red blood cells, and his hemoglobin concentration increased to 9.4 g/dL. Afterward, the patient underwent cerebral angiography and EMMA and was moved to the intensive care unit (ICU). One day later, the patient developed right-sided weakness and aphasia. Head CT displayed left middle cerebral artery (MCA) ischemic changes and SDH progression (Figure 2).

**Figure 3:**
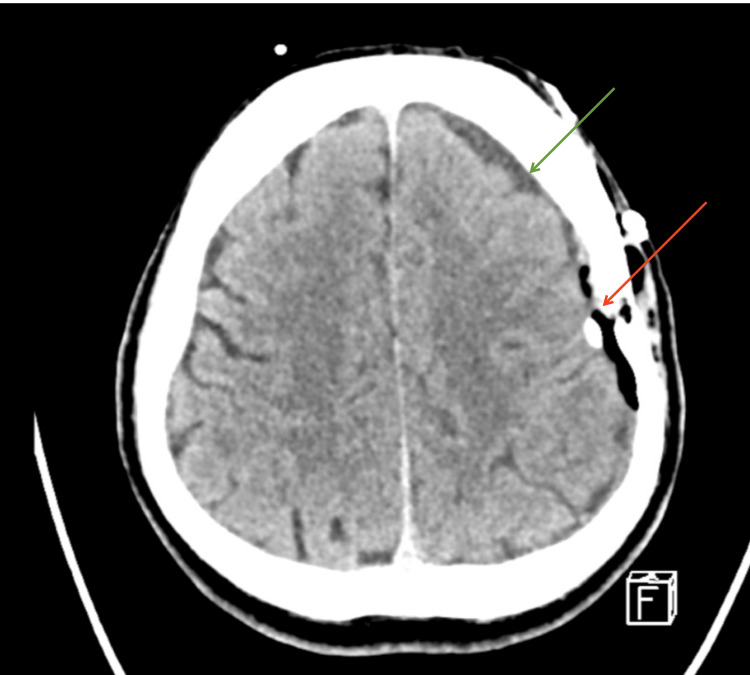
Post-burr hole evacuation CT show adequate evacuation, improvement of midline shift (green arrow), and pneumocephalus (red arrow).

The patient was taken for cerebral angiography with thrombectomy of the left M2 branch of the left MCA, burr hole evacuation of the left SDH, and subdural drain insertion. He was transferred back to the ICU for further management and monitoring. A subsequent head CT indicated a return of cerebral blood flow, adequate evacuation of the left SDH, and improvement of midline shift (Figure 3).

**Figure 4:**
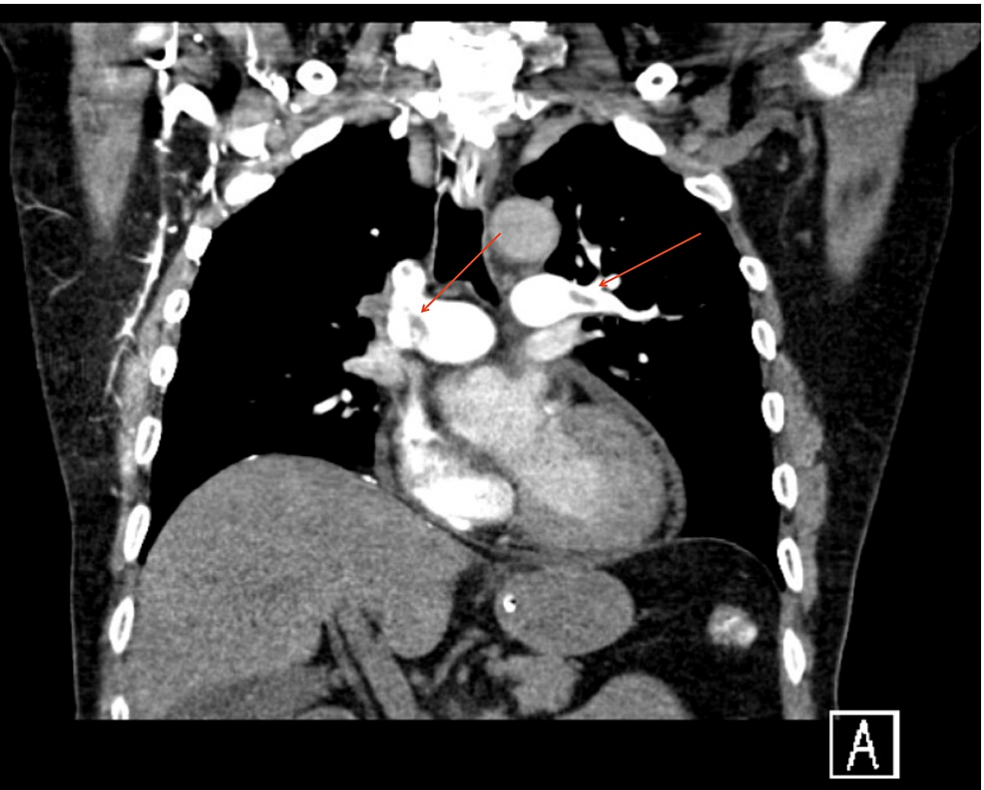
Chest CT angiogram show bilateral pulmonary embolism.

On further follow-up, the patient remained aphasic and developed right-side hemiplegia, for which he received rehabilitation therapy. Moreover, the patient was assessed for oral feeding tolerance by the ear-nose-and-throat team; examination revealed decreased laryngeal sensation and decreased refluxes; thus, he was unlikely to tolerate oral feeding. Furthermore, during the hospital stay, after nine days of EMMA, the patient developed a massive pulmonary embolism (Figure 4), and he was transferred back to the ICU. During the ICU stay, the patient was intubated for a long duration with failure to extubate; hence, a tracheostomy was performed. At discharge, the patient was aphasic, with right-sided hemiplegia and a gastrostomy feeding tube as well as a tracheostoma.

All procedures have their own risks and benefits, and the physician must make their decision based on the current evidence and their experience. This patient underwent EMMA to manage the acute on chronic SDH as it has low potential for complications; however, he developed ischemic stroke and aphasia as complications of EMMA that resulted in being hemiplegic and aphasic, which worsened his outcome. Moreover, the fact that the patient underwent surgery, immobility, and a prolonged hospital stay all contribute to the development of massive PE, which even worsens the patient outcome.

## Discussion

Subdural hematoma is an accumulation of blood between the dura mater and the arachnoid layer, which could be acute, subacute, or chronic [1]. Acute-on-chronic subdural hematoma is an acute hemorrhage on a pre-existing hematoma. CSDH is one of the most common neurosurgical conditions and presents with a wide range of symptoms [2]. In this case, the patient’s main complaint was headache, which is the most frequently reported symptom of patients with CSDH [2,9]. The associated symptoms were gait instability and vertigo; further symptoms may include the neurological deficit, tinnitus, seizure, or nausea and vomiting [2,10]. Hertha et al. reported a case of a 34-year-old female who presented with acute bilateral paraplegia and urinary retention, which were due to bilateral acute on chronic SDH [11].

Although falls and head trauma represent the greatest risk factors, other issues may be involved [2]. These factors include hypertension, bleeding diathesis, the use of anticoagulants, and cerebral atrophy, which is due to aging and/or chronic alcohol consumption [3,4,10]. Alcoholism is an important risk factor, not only due to its effect on cerebral atrophy but also due to its depressant effect, which increases the probability of falls and head trauma [10-12]. Despite the patient's denial of any head trauma, CSDH was a possibility for him due to his age and other medical issues.

Neuroimaging is crucial for the diagnosis and evaluation of CSDH; however, laboratory investigations are also important. The patient may accumulate a significant amount of blood in the subdural space before developing symptoms; therefore, a careful reading of the CBC may show a change in the concentration of hemoglobin and the hematocrit. As in this case, the patient’s CBC indicated a hemoglobin decrease, and no other bleeding sources were identified. Likewise, the accumulation of blood over time can result in consumptive coagulopathy, the first sign of which is a decreased platelet count [13].

The mainstay of the management of CSDH is surgical management. Symptomatic patients with a hematoma of 10 mm or more and/or a mass effect are candidates for surgical management [1,2]. The preferred option is burr-hole craniotomy, as it has the lowest risk and a low chance of recurrence [2,3]. In this case, EMMA was performed first; after that, the patient developed an ischemic stroke, with a thrombectomy implemented subsequently to manage the stroke with a burr hole craniotomy and drain insertion. Evidence suggests that using a drain helps further decrease the chance of recurrence [3].

Embolization of MMA is a new management option that lacks supporting evidence over surgical management; however, it is a safe option when used in conjunction with surgical management to decrease the recurrence rate [5,14]. Even so, it has some complications; treatment failure is the most common one, which can result in worsening of the hematoma and neurological deficits. Moreover, anatomic variation carries a risk of unintended embolization, which can result in blindness, stroke, and facial nerve palsy [5,6]. Gerstl et al. performed a systematic review of the complications of EMMA and discovered a 3.79% overall complication incidence with 1.33% neurological complications such as stroke, aphasia, and visual changes. Furthermore, they described 0.27% of cardiovascular complications, such as deep vein thrombosis and PE. Other complications include infections as well [15]. Despite the low chance of developing these types of complications, our patient developed neurological and cardiovascular issues in the form of ischemic stroke, aphasia, and PE.

Outcomes of CSDH are poorly documented, and the patient prognosis is largely dependent on their clinical condition at presentation [2]. Earlier diagnosis and intervention have better outcomes; however, the presence of comorbidities such as heart disease and renal failure has a great impact on prognosis [2,3]. In our case, diagnosis and intervention were delayed, as well as the complication of embolization, all of which impacted this patient’s outcome.

This presented case underscores the critical importance of recognizing and promptly addressing symptoms such as headaches and vertigo, particularly in older individuals with comorbidities. Physicians should remain vigilant when managing older patients with comorbidities, as these individuals may present with subtle yet significant neurological symptoms. Moreover, the red flags of headache and vertigo in the elderly, especially when accompanied by anemia, should be taken seriously. Therefore, delays in diagnosis and subsequent intervention can be avoided; in this case, such delays led to surgical dilemmas and complications, including ischemic stroke and pulmonary embolism. Furthermore, increasing awareness among healthcare professionals is crucial to ensuring timely intervention and minimizing the risk of complications. This case serves as a poignant reminder of the potential consequences of overlooking or delaying the diagnosis of acute-on-chronic SDH.

## Conclusions

To conclude, CSDH is a common neurosurgical condition; on the other hand, acute-on-chronic subdural hematoma is not common. Its presentation varies, and most of the patients present with headaches that typically worsen over the last few days. Many risk factors play a role in this condition, with head trauma being the most important as well as aging. Burr-hole craniotomy is the most effective management option because it has a low potential for complications and recurrence. On the other hand, EMMA is generally a safe procedure that has a small potential for significant complications that need to be studied more.
